# Supplementary material for: Molecular polarizability anisotropy of liquid water revealed by terahertz-induced transient orientation
Source: Nat Commun. 2018 May 30;9:2142. doi: 10.1038/s41467-018-04481-5 (PMC5976729; doi:10.1038/s41467-018-04481-5)
Supplement: Supplementary file 1 — Supplementary Information [file 41467_2018_4481_MOESM1_ESM.pdf]

Molecular polarizability anisotropy of liquid water revealed by terahertz-induced transient orientation

Zalden et al.

Molecular polarizability anisotropy of liquid water revealed by terahertz-induced transient orientation

Peter Zalden[1,2*+], Liwei Song[3,4*], Xiaojun Wu[3,5], Haoyu Huang[3], Frederike Ahr[3], Oliver D. Mücke[1,3], Joscha Reichert[1,6], Michael Thorwart[1,6], Pankaj Kr. Mishra[1,3], Ralph Welsch[3], Robin Santra[1,3,7], Franz X. Kärtner[1,3] and Christian Bressler[1,2]

1) Centre for Ultrafast Imaging CUI, University of Hamburg, 22761 Hamburg, Germany.
2) European XFEL, Holzkoppel 4, 22869 Schenefeld, Germany.
3) Center for Free-Electron Laser Science CFEL, Deutsches Elektronen-Synchrotron, 22607 Hamburg, Germany.
4) State Key Laboratory of High Field Laser Physics, Shanghai Institute of Optics and Fine Mechanics, Chinese Academy of Sciences, 201800 Shanghai, China.
5) School of Electronic and Information Engineering, Beihang University, 100191 Beijing, China.
6) I. Institut für Theoretische Physik, University of Hamburg, Jungiusstr. 9, 20355 Hamburg, Germany.
7) Department of Physics, University of Hamburg, Jungiusstr. 9, 20355 Hamburg, Germany

*These authors contributed equally
+Corresponding author: peter.zalden@xfel.eu

Supplementary Note 1: Experimental setup and data analysis

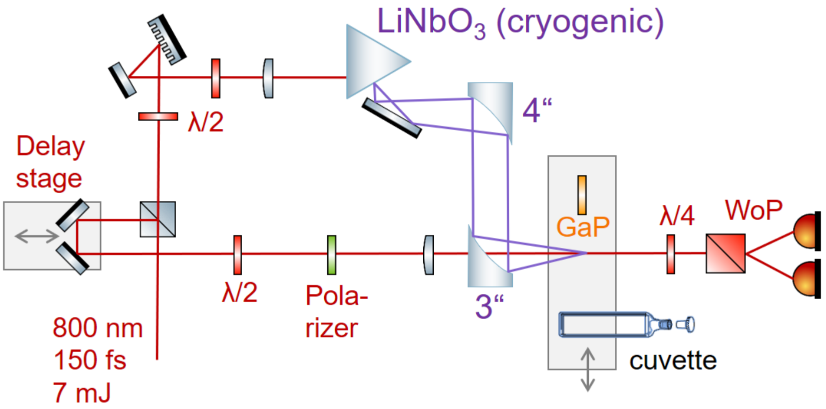

Supplementary Figure 1: Schematic of the experimental setup: The optical beam (red line) is split into a weak probe and a strong pump beam, which are used to measure the optical birefringence with balanced photodiodes A and B, sensitive to the probe intensity polarized parallel to the electric field of the THz pulse and perpendicular to it. The pump beam is used to generate THz pulses from $LiNbO_3$.

This section contains a summary of the experimental setup and of the simulations performed to evaluate the local Kerr effect inside the cuvette and in the liquid. The experimental setup, depicted in Supplementary Figure 1, utilizes optical pulses from a Ti:Sapphire chirped-pulse laser amplifier with fundamental wavelength of 800 nm, 150 fs pulse duration and 7 mJ pulse energy to generate THz pulses by optical rectification in $LiNbO_3$. The optical pulse-fronts are tilted by a grating to fulfill the phase-matching condition. The THz pulses generated from this source are de-magnified using two off-axis parabolic mirrors with 4" and 3" focal lengths. In the image plane, the pulses are characterized by electro-optic (EO) sampling using a 50 µm-thick <110>-cut GaP and a 200 µm-thick <110>-cut ZnTe crystal. The electric field waveform consists of a single cycle with peak electric field strength of 510 kV/cm and 0.25 THz center frequency. The THz beam diameter in the focus is one millimeter – an order of magnitude larger than the optical probe spot. For the measurement of liquid samples, Spectrosil® (synthetic fused silica) cuvettes are used with 1 cm diameter aperture and two 1.2 mm-thick windows enclosing a 0.2 mm-thick sheet of liquid. Only for carbon disulfide (CS_2), due to its low THz absorption coefficient, a cuvette of 2 mm inner thickness was used. To measure the

optical birefringence, the polarization of the probe beam is tilted by 45° with respect to the THz electric field polarization. A Kerr effect time trace is recorded by scanning the delay between the pump (500 Hz repetition rate) and the probe (1 kHz), reading the pump-induced modulation detected by the balanced photodiodes using a lock-in amplifier. The data are normalized to the signal obtained when chopping the probe beam at 500 Hz, with the respective sample in the optical beam path.

The data extractions start from the incoming electric-field waveform of the THz pulse $E(t)$, known in absolute units from EO sampling. Let z be the propagation direction of the THz pulse, perpendicular to the windows of the cuvette. For every step Δz (positive when propagating in the same direction as the probe beam), the electric field pulse is Fourier transformed and propagated by multiplying $\exp(i\omega\Delta z \times (n(\omega) - n_0)/c)$, where $n(\omega)$ is the complex refractive index of the specific material in the THz regime, known from TDS, and n_0 is the (real) optical refractive index at the probe wavelength. When the pulse reaches an interface, it splits into a reflected and transmitted wave with its field strength modified by the reflection and transmission coefficients, $\left(n_1(\omega) - n_2(\omega)\right)/\left(n_1(\omega) + n_2(\omega)\right)$ and $2n_1(\omega)/\left(n_1(\omega) + n_2(\omega)\right)$, respectively. As an example, the propagation of a THz pulse through a cuvette filled with water is shown in Supplementary Figure 2. In case of water, due to its strong absorption in the THz regime, the field strength is reduced significantly upon passing the water layer.

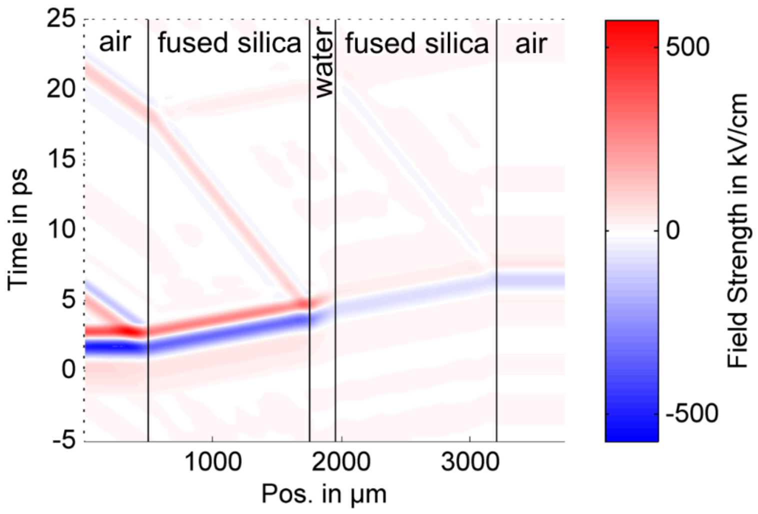

Supplementary Figure 2: Propagation of the single-cycle THz pulse through a cuvette, filled with water. The vertical lines indicate the interfaces between the two 1.2 mm-thick fused silica windows and the 0.2 mm-thick sheet of water. The THz pulse propagates from left to right. The vertical axis denotes the pump-probe delay, i.e., $\Delta t = \left(v_{\mathrm{THz}} - v_{\mathrm{opt}}\right)/c \times t$, where v_{THz} and v_{opt} are the group velocities of each material in the THz and optical regimes, respectively.

Applying the Kerr effect model, supplementary equations (1&2), to the simulated electric field data, one can exactly calculate the expected Kerr effect of the cuvette, considering absorption and reflection losses from the cuvette. This background signal is depicted as purple curve in Supplementary Figures 3&4 for illustration. It is then subtracted from the data while refining the Kerr coefficients of the liquid under investigation. The exact agreement of the model and experimental data for benzene as well as the alcohols confirms the accuracy of this approach.

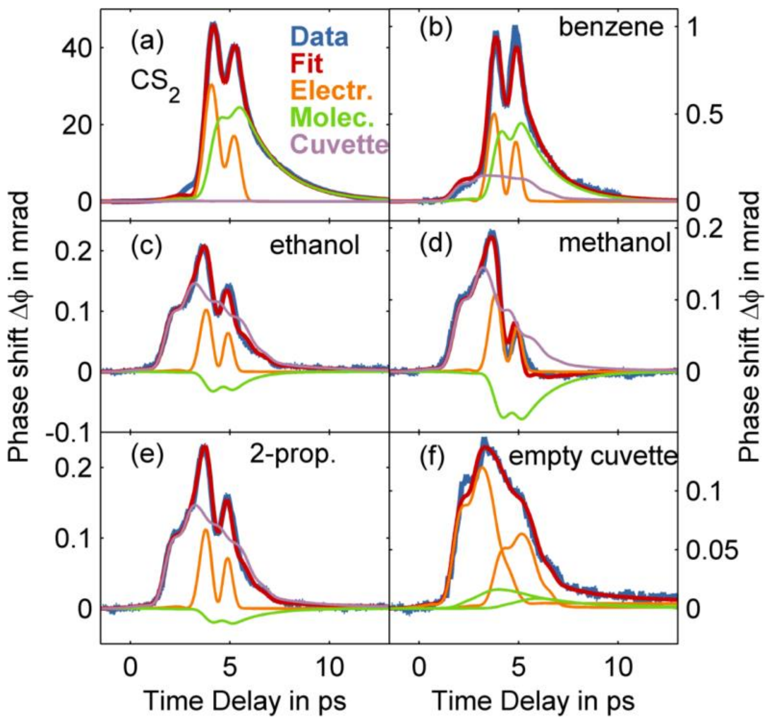

Supplementary Figure 3: The raw Kerr traces (blue curves) shown before subtracting the background from the cuvette (purple curves) as calculated for each sample (a)-(e) based on the known Kerr coefficient obtained from the refinement of an empty cuvette (f). Additionally, the red curves represent the refined model for the sum of electronic (orange curves) and molecular (green curves) Kerr effects. In (f), the two curves each correspond to the contribution of the front (early) and back (late) windows of the cuvette.

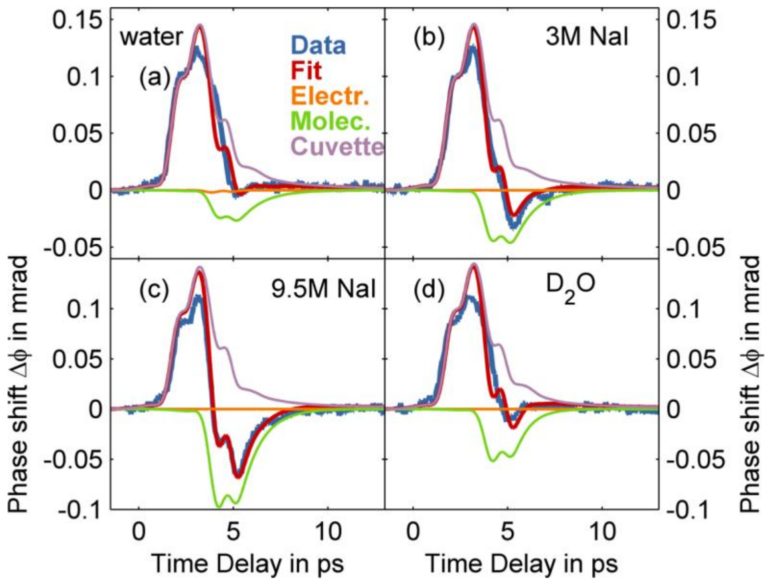

Supplementary Figure 4: The raw Kerr traces (blue curves) shown before subtracting the background from the cuvette (purple curves) as calculated for each sample (a)-(d) based on the known Kerr coefficient obtained from the refinement of an empty cuvette.

Supplementary Note 2: Field-strength dependence measurements of the Kerr effect

Supplementary Figure 5(a) shows the EO sampling curves as function of the pump energy on the $LiNbO_3$ crystal. From these curves we derive the field strength reached at each pump fluence. Supplementary Figure 5(b-d) shows Kerr effect measurements on the empty cuvette, methanol and water for different fluences of 7.4 mJ, 5 mJ and 3 mJ, respectively. The numbers shown next to the curves in Supplementary Figure 5(b-d) mark the time delay at which the values were extracted to determine the fluence dependence summarized in Supplementary Figure 5(e). It shows the induced birefringence as function of the electric field in the THz pulse together with linear and quadratic fits. The relation $\Delta\varphi \propto E^2$ is clearly observed with a slight tendency toward a higher exponent.

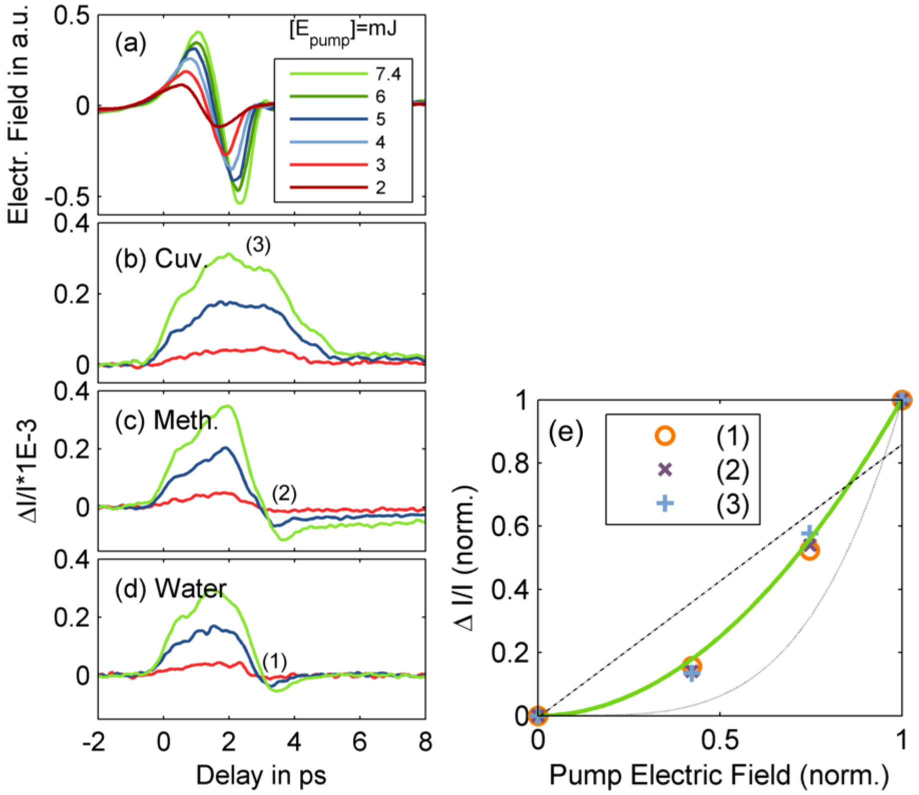

Supplementary Figure 5: Field-dependence of the THz-induced birefringence. (a) depicts the EO sampling traces for various pump fluences of the IR beam used to generate the THz pulses. (b-d) show the Kerr effect traces of an empty cuvette, methanol and water, respectively, at three different pump conditions (7.4 mJ, 5 mJ and 3 mJ). Numbers in brackets denote a specific feature of the data, whose mean amplitude was determined and plotted against the electric field strengths, extracted from (a) and shown in (e). All features scale with the square of the THz electric field (green curve). The best fit exponent is 2.3±0.2. Dashed lines show linear and quartic scaling for comparison, which can be ruled out.

Supplementary Note 3: Time-domain spectroscopy

THz time-domain spectroscopy (TDS) was performed using a commercial fiber-laser and antenna-based THz-generation system operating at MHz repetition rates. The TDS scans were performed under nitrogen purging and for every sample the empty cuvette was sealed and measured as reference. Two cuvettes of different thickness were used to adjust for the absorption in each liquid. Both cuvettes are made of two flat fused silica windows, each of thickness $d' = 1.2$ mm. In the thick cuvette with $d = 2$ mm path length, all backreflections can be removed by temporal windowing, but for the thinner 200 µm-thick cuvettes, a transfer function approach must to be employed, which considers the backreflections inside the sample volume,

$$\frac{E_{\mathrm{cuv}}(\omega)}{E_{\mathrm{mty}}(\omega)} = \frac{16N'^2 \times \exp((N'-1)2i\omega d'/c)}{\exp(2i\omega d/c) \times \left(1-N'^2\right)^2 - (N'+1)^4} \tag{1}$$

where $E_{\mathrm{mty}}(\omega)$ and $E_{\mathrm{cuv}}(\omega)$ are the complex-valued Fourier transformed TDS traces of the empty beam path and the empty cuvette, respectively. N' is the refractive index of the cuvette material (fused silica), which can be determined numerically for each frequency value in the data. Similarly,

$$\frac{E_{\mathrm{liq}}(\omega)}{E_{\mathrm{cuv}}(\omega)} = N\frac{\exp(i\omega d/c) \times (N'-1)^2 - \exp(-i\omega d/c) \times (N'+1)^2}{\exp(i\omega dN/c) \times (N'-N)^2 - \exp(-i\omega dN/c) \times (N'+N)^2} \tag{2}$$

for the filled cuvette, $E_{\mathrm{liq}}(\omega)$, with refractive index N of the liquid inside the cuvette, again considering backreflections through the liquid. Data obtained in this way are depicted in Supplementary Figures 6&7. In Supplementary Figure 6 it is clearly seen that the liquids under investigation possess quite different absorption coefficients with the lowest absorption observed in CS_2 and the highest in water. The latter fact makes it particularly difficult to excite sufficiently thick layers of water by strong THz electric fields. Furthermore, Supplementary Figure 7 shows the THz dielectric function of water and methanol for different concentrations of NaI.

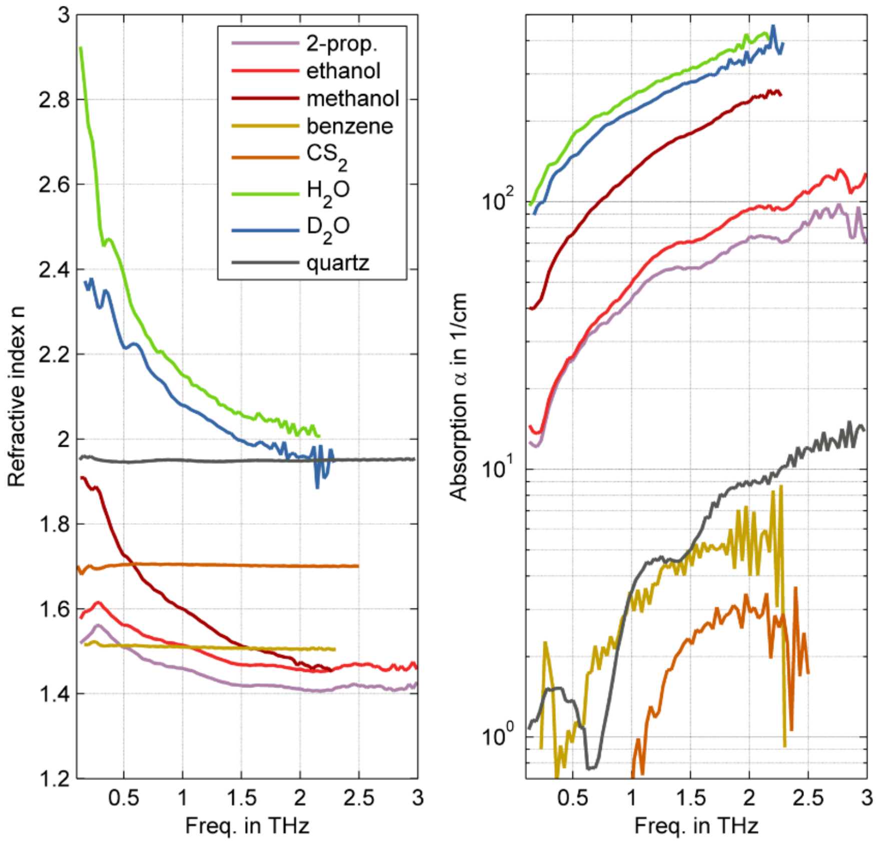

Supplementary Figure 6: THz complex refractive index of the liquids, whose THz Kerr effect is determined in the main text. The spectra are obtained by time-domain spectroscopy (TDS). The large absorption coefficient of some liquids, water and methanol must be considered for the calculation of the Kerr coefficient. Periodic oscillations in the data are caused by interferences inside the cuvette, which are not fully subtracted by the transfer function formalism described here.

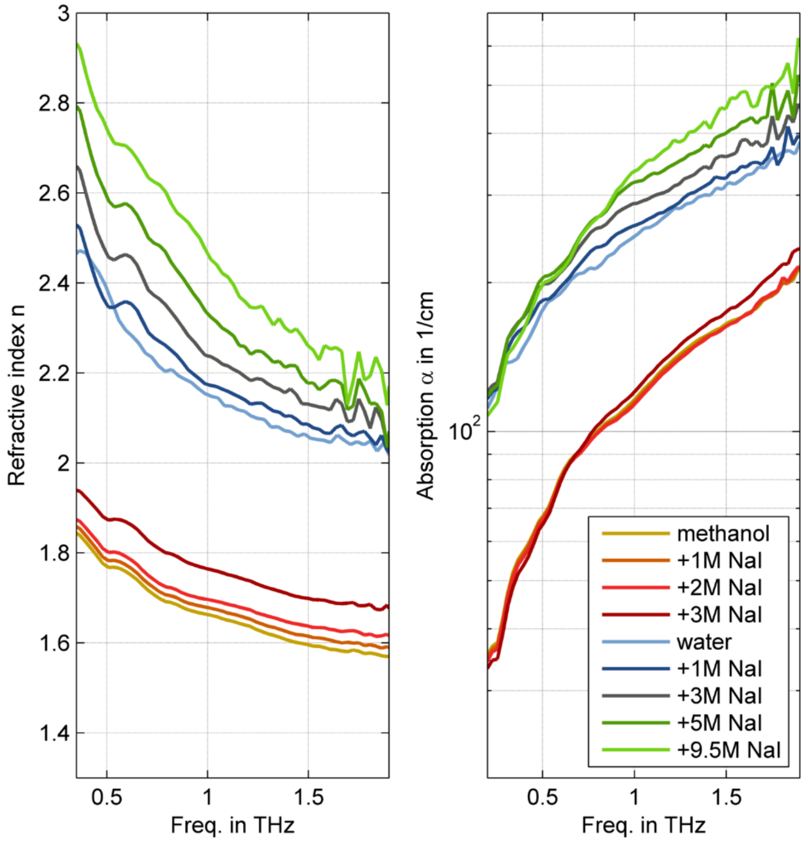

Supplementary Figure 7: Complex refractive index data of water and methanol at different concentrations of NaI, obtained using TDS. In water and methanol, there is a clear trend of increasing refractive index. The absorption increases continuously with NaI concentration in water only at frequencies above about 1 THz.

Supplementary Note 4: Time-domain model for the Kerr effect based on the Langevin equation

Theory of Kalmykov and Coffey

A theoretical description for dynamic Kerr signals of polar, asymmetric-top molecules in a solvent has been given by Kalmykov and Coffey[1,2]. In their approach, the emergence of birefringence in response to strong electric fields is treated based on rotational Brownian motion of single molecules. Specifically, a perturbing electric field seeks to rotate molecules on basis of their permanent dipole moments and (hyper-) polarizabilities, while thermal fluctuations aim to lead the system back towards an equilibrium distribution. This interplay is captured by an Euler-Langevin equation in the overdamped (Debye) and dilute (non-interacting) limit, which is used to derive a hierarchy of differential recurrence relations for the appropriate statistical moments of the problem. In this case, these are given by averages of Wigner's D functions $D^{j}_{m,n}(t)$[3] which form an orthonormal basis set for the construction of the relevant quantities. For comparably low electric field strengths the hierarchy can be truncated early, leading to a simple description via two coupled vector-differential equations

$$\frac{d}{dt}\boldsymbol{c}_1(t) = \hat{A}_1\boldsymbol{c}_1(t) + E(t)\boldsymbol{B}_1 \tag{3}$$

$$\frac{d}{dt}\boldsymbol{c}_2(t) = E(t)\hat{Q}\boldsymbol{c}_1(t) + \hat{A}_2\boldsymbol{c}_2(t) + E^2(t)\boldsymbol{B}_2. \quad (4)$$

Here, the vectors $\boldsymbol{c}_1(t)$ and $\boldsymbol{c}_2(t)$ carry averages of the first and second Wigner functions,

$$\boldsymbol{c}_1(t) = \begin{pmatrix} \langle D^1_{0,-1}\rangle(t) \\ \langle D^1_{0,0}\rangle(t) \\ \langle D^1_{0,1}\rangle(t) \end{pmatrix}, \boldsymbol{c}_2(t) = \begin{pmatrix} \langle D^2_{0,-2}\rangle(t) \\ \langle D^2_{0,-1}\rangle(t) \\ \langle D^2_{0,0}\rangle(t) \\ \langle D^2_{0,1}\rangle(t) \\ \langle D^2_{0,2}\rangle(t) \end{pmatrix}. \quad (5)$$

Their time-evolution is determined by the coefficients of the rotational diffusion tensor D_{ij} and the response to the perturbing electric field $E(t)$. The latter is given by the permanent dipole moment $\boldsymbol{\mu}$ and the elements of the electric polarizability tensor ε_{ij} of a molecule. Hyperpolarizabilities are neglected here. In our case, the molecular reference frame xyz is chosen in such a way that the rotational diffusion tensor is diagonal and encoded within

$$\Delta = \frac{D_{zz}}{D_{xx}+D_{yy}} - \frac{1}{2}, \quad (6)$$

$$\tau_D = \frac{1}{D_{xx}+D_{yy}}, \quad (7)$$

$$\Xi = \frac{D_{xx}-D_{yy}}{D_{xx}+D_{yy}}. \quad (8)$$

For molecules with rotational symmetry around the z-axis, such as CS_2 and benzene, $\Xi = 0$.

The matrices $\hat{A}_1$ and $\hat{A}_2$ are then related to relaxation back towards the equilibrium distribution and read

$$\hat{A}_1 = -\tau_D^{-1}\begin{pmatrix} 1+\Delta & 0 & \Xi/2 \\ 0 & 1 & 0 \\ \Xi/2 & 0 & 1+\Delta \end{pmatrix}, \quad (9)$$

$$\hat{A}_2 = -\tau_D^{-1}\begin{pmatrix} 3+4\Delta & 0 & \Xi\sqrt{3/2} & 0 & 0 \\ 0 & 3+\Delta & 0 & 3\Xi/2 & 0 \\ \Xi\sqrt{3/2} & 0 & 3 & 0 & \Xi\sqrt{3/2} \\ 0 & 3\Xi/2 & 0 & 3+\Delta & 0 \\ 0 & 0 & \Xi\sqrt{3/2} & 0 & 3+4\Delta \end{pmatrix}, \quad (10)$$

where the diagonal entries give rise to relaxation times of the order of τ_D and $\tau_D/3$, respectively, if Ξ and Δ can be neglected. The vector $\boldsymbol{B}_1(t)$ and the matrix $\hat{Q}$ relate to the impact of the permanent dipole moment

$$\hat{Q} = \frac{\sqrt{3}}{10\tau_D k_B T}\begin{pmatrix} \mu^-(3+4\Delta) - \mu^+\Xi & \sqrt{2}\mu_z\Xi & 0 \\ 3\mu_z & [\mu^-(3+2\Delta) - 2\mu^+\Xi]/\sqrt{2} & \mu_z\Xi \\ \sqrt{3/2}(\mu^-\Xi - \mu^+) & 2\sqrt{3}\mu_z & \sqrt{3/2}(\mu^- - \mu^+\Xi) \\ \mu_z\Xi & [2\mu^-\Xi - \mu^+(3+2\Delta)]/\sqrt{2} & 3\mu_z \\ 0 & \sqrt{2}\mu_z\Xi & \mu^-\Xi - \mu^+(3+4\Delta) \end{pmatrix}, \quad (11)$$

$$\boldsymbol{B}_1 = \frac{1}{3\sqrt{2}\tau_D k_B T}\begin{pmatrix} \mu^-(1+\Delta) - \mu^+\Xi/2 \\ \sqrt{2}\mu_z \\ -\mu^+(1+\Delta) + \mu^-\Xi/2 \end{pmatrix}, \tag{12}$$

where $\mu^\pm = \mu_x \pm i\mu_y$. Finally, the vector $\boldsymbol{B}_2(t)$ gives the coupling to the electric polarizability

$$\boldsymbol{B}_2 = \frac{1}{10\sqrt{6}\tau_D k_B T}\begin{pmatrix} (\varepsilon_{xx} - 2i\varepsilon_{xy} - \varepsilon_{yy})(3+4\Delta) - (\varepsilon_{xx} + \varepsilon_{yy} - 2\varepsilon_{zz})\Xi \\ 2(\varepsilon_{xz} - i\varepsilon_{yz})(3+\Delta) - 3(\varepsilon_{xz} + i\varepsilon_{yz})\Xi \\ \sqrt{6}[2\varepsilon_{zz} - \varepsilon_{xx} - \varepsilon_{yy} + \Xi(\varepsilon_{xx} - \varepsilon_{yy})] \\ -2(\varepsilon_{xz} + i\varepsilon_{yz})(3+\Delta) + 3(\varepsilon_{xz} - i\varepsilon_{yz})\Xi \\ (\varepsilon_{xx} + 2i\varepsilon_{xy} - \varepsilon_{yy})(3+4\Delta) - (\varepsilon_{xx} + \varepsilon_{yy} - 2\varepsilon_{zz})\Xi \end{pmatrix}. \tag{13}$$

In a Kerr measurement, the perturbing electric field is assumed to occur along the axis Z in the laboratory coordinate system spanned by the three axes XYZ. The birefringence is then measured between the axes X and Z with an optical probe pulse. It can be derived from the measured optical anisotropy $\Delta\alpha(t) = \langle \alpha_{ZZ} - \alpha_{XX}\rangle(t)$ by way of the Lorenz-Lorentz equation as

$$\Delta n(t) \approx \frac{2\pi\rho_0}{n_s}\Delta\alpha(t), \tag{14}$$

with the number density ρ_0, the refractive index of the solvent n_s and formulated here for a polarizability volume $\alpha = \alpha'/4\pi\varepsilon_0$ [4,5]. The connection between $\Delta\alpha(t)$ and Supplementary equations (3) and (4) is given by a dot product

$$\Delta\alpha(t) = \boldsymbol{a}_2 \cdot \boldsymbol{c}_2(t), \tag{15}$$

where $\boldsymbol{a}_2$ contains the spherical components of the body-fixed optical polarizability tensor $\hat{\alpha}_0$ and reads

$$\boldsymbol{a}_2 = \sqrt{3/8}\begin{pmatrix} \alpha_{xx} - \alpha_{yy} + 2i\alpha_{xy} \\ 2(\alpha_{xz} + i\alpha_{yz}) \\ \sqrt{6}[\alpha_{zz} - \mathrm{Tr}[\hat{\alpha}]/3] \\ 2(-\alpha_{xz} + i\alpha_{yz}) \\ \alpha_{xx} - \alpha_{yy} - 2i\alpha_{xy} \end{pmatrix}. \tag{16}$$

The vector $\boldsymbol{a}_2$ contains anisotropies of the optical polarizability and defines the dimension and overall size of the resulting signal. In fact, its central component can be rewritten as $\Delta\alpha_0 = \alpha_{0,\|} - \alpha_{0,\perp} = \alpha_{zz} - (1/2)(\alpha_{xx} + \alpha_{yy})$ and multiplies to the central components of both $\boldsymbol{c}_1$ and $\boldsymbol{c}_2$. If we choose the molecular z-axis to coincide with the permanent dipole moment, these elements give direct measures for orientation and alignment of the molecules as

$$\langle D^1_{0,0}\rangle(t) = \langle\cos(\theta)\rangle(t) \tag{17}$$

$$\langle D^2_{0,0}\rangle(t) = \frac{1}{2}\left(\langle 3\cos^2(\theta) - 1\rangle(t)\right). \tag{18}$$

Both relations follow from a general property of the D functions, specifically $D^j_{0,0} = P_j(\cos(\theta))$, where P_j is the Legendre polynomial of rank j, and θ the Euler angle between the molecular z-axis and the laboratory Z-axis [2,3]. For the case of isotropic rotational diffusion, where $D_{xx} = D_{yy} = D_{zz} = D$ such that $\Xi = \Delta = 0$, we can formally solve the theory of Supplementary equations (3) and (4). Assuming isotropic initial conditions, where $\boldsymbol{c}_1(0) = 0$ and $\boldsymbol{c}_2(0) = 0$, as well as choosing the molecular z-axis to coincide with the permanent dipole moment, we obtain

$$\Delta\alpha(t) = \frac{1}{5\tau_D}\left[\frac{3}{4}\frac{\Delta\alpha_0^+\Delta\varepsilon_0^+}{k_BT} + \frac{\Delta\alpha_0\,\Delta\varepsilon_0}{k_BT}\right]\int_0^t du\, E^2(u)e^{-3\frac{(t-u)}{\tau_D}} + \frac{1}{5\tau_D^2}\frac{\Delta\alpha_0\,\mu_z^2}{(k_BT)^2}\int_0^t du\int_0^u ds\left[E(u)e^{-3\frac{(t-u)}{\tau_D}}\right]\left[E(s)e^{-\frac{(u-s)}{\tau_D}}\right], \tag{19}$$

where we defined $\Delta\alpha_0^+ = \alpha_{xx} - \alpha_{yy}$ and $\Delta\varepsilon_0^+ = \varepsilon_{xx} - \varepsilon_{yy}$ as well as $\Delta\varepsilon_0 = \varepsilon_{zz} - (1/2)(\varepsilon_{xx} + \varepsilon_{yy})$ in accordance with $\Delta\alpha_0$. Supplementary equation (19) renders two distinct behaviors: the first term induces a unipolar signal which depends on the anisotropies of the electric polarizability and the second creates a bipolar term that depends on the permanent dipole moment. For molecules with zero permanent dipole moment, we expect a unipolar contribution consistent with the results for benzene and CS_2 in the main text. For molecules with non-zero permanent dipole moment, the second term contributes as well and we expect an interplay between both contributions. The latter may be a particularly important component for the measurement on water and the alcohols. As such, we implement the theory of Supplementary equations (3) and (4) for the case of liquid water in the next section.

Implementation for liquid water

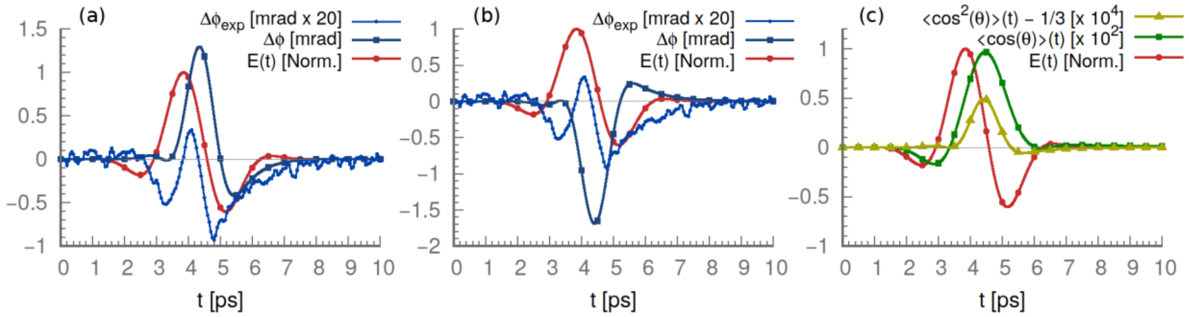

Supplementary Figure 8: Phase shift $\Delta\phi(t)$ for the case of liquid water obtained from Supplementary equations (3) and (4) (dark blue lines with squares) versus the experimental result $\Delta\phi_{\text{exp}}(t)$ (light blue dash-dotted lines). Panel (a) shows the theoretical prediction for the optical polarizability of Supplementary equation (21), while panel (b) shows the result for the polarizability of Supplementary equation (22). The experimental result has been scaled by a factor of 20 to account for absorption and to enable a rough comparison. Panel (c) shows a measure for orientation $\langle\cos(\theta)\rangle(t)$ (solid green line with squares) and alignment $\langle\cos^2(\theta)\rangle(t)$ (dotted yellow line with triangles) obtained from Supplementary equations (17) and (18). The perturbing electric field $E(t)$ is modelled as the Gaussian of Supplementary equation (23) and is shown normalized in all panels (solid red line with circles).

Supplementary equations (3) and (4) have been evaluated numerically in Supplementary Figure 8. To do so, we extracted the diagonal elements of the rotation diffusion tensor from previous molecular dynamics simulations[6], specifically

$$\hat{D} = \begin{pmatrix} 0.211 & 0 & 0 \\ 0 & 0.114 & 0 \\ 0 & 0 & 0.272 \end{pmatrix} 1/\text{ps}. \tag{20}$$

Here, we assumed that the principal axes coincide with the principal axes of inertia such that the z-direction points in the direction of the permanent dipole moment, x spans the H-H-direction and y

points out of the molecular plane. The resulting timescale is then given as $\tau_D \approx 3$ ps where $\tau_D/3 \approx 1$ ps. We note in passing, that a different choice of $\widehat{D}$ (see, e.g., refs. [7–9]) does not lead to a significant change in the overall theoretical result. The polarizability tensors for water are assumed to be diagonal in the molecular frame chosen. To enable a comparison we use the same values as those in Supplementary Note 5. Specifically, we utilize values for water vapor[10]

$$\hat{\alpha}_0^{(1)} = \begin{pmatrix} 1.626 & 0 & 0 \\ 0 & 1.286 & 0 \\ 0 & 0 & 1.495 \end{pmatrix} \text{Å}^3 , \tag{21}$$

which has a $\Delta\alpha_0^{(1)} \approx 0.04\ \text{Å}^3 > 0$ and a second set derived from coupled cluster theory calculations[11]

$$\hat{\alpha}_0^{(2)} = \begin{pmatrix} 1.375 & 0 & 0 \\ 0 & 1.442 & 0 \\ 0 & 0 & 1.321 \end{pmatrix} \text{Å}^3 , \tag{22}$$

which features a $\Delta\alpha_0^{(2)} \approx -0.09\ \text{Å}^3 < 0$. The polarizability in the THz regime together with the permanent dipole moment governs the response to the THz pulse. As results for the static polarizability tensor of water do not deviate much from Supplementary equations (21) and (22) (see, e.g., [12]) we assume the electric polarizability given by the ε_{ij} to be equal to the optical polarizability values. The permanent dipole moment of a water molecule is given as $\boldsymbol{\mu} = \mu \boldsymbol{e}_z$ with $\mu = 2.95$ D, which is an appropriate value in the liquid phase[13,14]. The THz pulse $E(t)$ is modelled as in Supplementary equation (24) with

$$E(t) = A \cdot E_0 \cdot \cos(\omega_c \mathrm{t} + \varphi) e^{-\frac{(t_p - t)^2}{2\sigma^2}}, \tag{23}$$

where we use slightly different values and set $\omega_c = 2\pi \cdot 0.3$ THz and the phase to $\varphi = -\pi/4$. We center the pulse at $t_p = 4.2$ ps and set its width to $\sigma = 1$ ps. The prefactor $A \approx 1.1$ normalizes the pulse such that its maximum gives $E_0 = 510$ kV/cm, in accordance with the experimental parameter. For this value $\mu E_0/k_B T \approx 0.1$ at room temperature, which is appropriate for the low-energy theory we consider. Note, however, that we do not take absorption effects into account in this way. Finally, we evaluated the theory at room temperature and chose the number density equal to that of liquid water as $\rho_0 = (1/30)\ \text{Å}^{-3}$. In order to compare to experimental values, the phase-shift has been obtained by $\Delta\phi(t) = (2\pi L/\lambda)\Delta n(t)$, where we used $L = 0.2$ mm as the thickness of the liquid sheet in the cuvette and the probing wavelength $\lambda = 800$ nm. As initial conditions we assumed isotropy, i.e. $\boldsymbol{c}_1(0) = 0$ and $\boldsymbol{c}_2(0) = 0$.

Supplementary Figure 8 shows the result for the polarizabilities of Supplementary equations (21) and (22) in contrast to the experimental results. The dynamics are dominated by the permanent dipole moment contribution entering through the dynamic vector $\boldsymbol{c}_1(t)$. The reason for this lies in the fact that the polarizability of water is nearly isotropic such that $\Delta\varepsilon_0$ and $\Delta\varepsilon_0^+$ are small. Therefore, the resulting curves show a bipolar behavior as expected from the second term in the isotropic solution of Supplementary equation (19) as the contribution related to the first term is negligible. A direct comparison of prefactors also yields $(\Delta\alpha_0/k_B T)/(\mu^2/(k_B T)^2) \approx 10^{-4}$.

The results in Supplementary Figure 8(a&b) show only a rough qualitative agreement to the experimental results with somewhat similar relaxation behavior and similar structure but marked differences in both the height as well as the exact position of the minima and maxima. This implies that our model is insufficient to describe the exact Kerr dynamics in the case of liquid water. This observation matches well with the fact that the isotropic solution in Supplementary equation (19) cannot be readily fitted to the experimental data, unless one of the relaxation times becomes much smaller than the other. However, the net negative and positive effects obtained when calculating the

Kerr effect using a negative $\Delta\alpha_0^{(2)}$ or positive $\Delta\alpha_0^{(1)}$ shows that $\Delta\alpha$ must be negative to explain a net negative Kerr effect. In addition, we also note that the average peak electric field in the 0.2 mm-long water layer is only around 150 kV/cm, reducing the ratio of peak Kerr effects in experiment and simulation to less than 2. Finally, Supplementary Figure 8(c) shows the resulting measures for orientation and alignment. As predicted, the electric field pulse couples strongly to the permanent dipole moment and orients the molecules while the alignment dynamics follows this profile as well. In addition, the resulting behavior agrees well with the simulation results obtained in Supplementary Note 5 (Figure 2 of the main text).

Despite the rough qualitative agreement, however, these results evidently call for a more involved treatment. In particular, the Debye approximation assumes strong damping and excitation energies not higher than the low GHz range, both insufficient for small molecules such as water and the higher frequency of the pump pulse[2]. Additionally, another particularly strong limitation is given by the assumption of a dilute liquid and, thus, non-interacting molecules. It is well known that water and alcohols interact through the formation of H-bonds which may strongly impact the rotation dynamics underlying the present theory. Lastly, the theory also neglects effects from hyperpolarizabilities such that, for instance, an instantaneous electronic contribution to the signal cannot be described. Other potential sources of errors may include quantum, memory and conduction effects which are neglected a priori in the present theory.

Supplementary Note 5: Molecular Dynamics Simulation

Computational Details

To investigate the THz Kerr effect on liquid water computationally, molecular dynamics (MD) simulations of bulk water were performed employing the rigid TIP4P/2005 force field[15] under periodic boundary conditions using the Large-scale Atomic/Molecular Massively Parallel Simulator (LAMMPS)[16]. A simulation box of 256 water molecules at a density of 1 g/cm^3 was thermally equilibrated at 300 K using the Nose-Hoover thermostat[17]. After the equilibration, 5000 initial atomic configurations and velocities were generated from a long MD trajectory performed under canonical (NVT) conditions. These different sets of initial conditions were sampled with a time interval of 1 ps to avoid artificial correlations between each sampled phase space point. Each set of initial conditions was then propagated in the presence of a THz pulse for 6 ps. All trajectories were propagated using the velocity-Verlet algorithm with 1 fs time step along with the SHAKE algorithm[18] to fix the intramolecular bonds and angles. A cut-off radius of 15 Å was used for the calculation of the short-range Lennard-Jones interactions and Particle-Particle-Particle-Mesh technique was used to calculate the electrostatic interaction.

The THz pump pulse employed in the simulations is given by

$$\boldsymbol{E}(t) = A\exp\left(-\frac{(t-t_0)^2}{2\sigma^2}\right)\boldsymbol{u}_z\cos(\omega_c t + \phi) \qquad (24)$$

with $\sigma = 800$ fs. The central photon frequency considered is $\omega_c = 2\pi \times 0.39$ THz (13 cm^{-1}), which results in a full cycle long pulse. The polarization direction of the electric field is $\boldsymbol{u}_z = (0,0,1)$ and ϕ is the carrier-envelope phase, which is set to $\pi/2$. The maximum electric field amplitude $A = 0.05$ corresponds to a power per unit area of 3.387×10^{10} W/cm^2.

The time-dependent refractive indices parallel ($n_\parallel(t)$) and perpendicular ($n_\perp(t)$) to the THz pulse polarization direction can be obtained from the polarizability of the sample as[5]

$$n_{\parallel}(t) = \sqrt{1 + 4\pi\left(\frac{N}{V}\right)\langle\alpha_{\parallel}\rangle(t)} \tag{25}$$

$$n_{\perp}(t) = \sqrt{1 + 4\pi\left(\frac{N}{V}\right)\langle\alpha_{\perp}\rangle(t)} \tag{26}$$

Here, the average polarizability per molecule $\langle\alpha\rangle$ is obtained from M MD trajectories with N molecules each, as

$$\langle\alpha\rangle(t) = \frac{1}{MN}\sum_{j=1}^{MN} R^T\left(\Phi_j(t), \theta_j(t), \Psi_j(t)\right)\alpha^G R\left(\Phi_j(t), \theta_j(t), \Psi_j(t)\right) \tag{27}$$

with the gas-phase polarizability $\alpha^G = \begin{pmatrix} 1.626 & 0 & 0 \\ 0 & 1.286 & 0 \\ 0 & 0 & 1.495 \end{pmatrix}$ Å^3[10]. The rotation matrices $R\left(\Phi_j(t), \theta_j(t), \Psi_j(t)\right)$ rotate the j-th molecular frame (z-axis along the molecular dipole, x-axis along the H-H axis and y-axis perpendicular to the molecular plane) to the simulation-box frame using the three Euler angles Φ_j, θ_j, Ψ_j. By assuming a uniform distribution for Φ and Ψ, one obtains

$$\langle\alpha_{\parallel}\rangle(t) = \hat{\alpha}^G + \left(\alpha_{\parallel}^G - \alpha_{\perp}^G\right)\left(\langle\cos^2(\theta)\rangle(t) - \frac{1}{3}\right) \tag{28}$$

$$\langle\alpha_{\perp}\rangle(t) = \hat{\alpha}^G - \left(\alpha_{\parallel}^G - \alpha_{\perp}^G\right)\left(\langle\cos^2(\theta)\rangle(t) - \frac{1}{3}\right) \tag{29}$$

where $\hat{\alpha}^G = 1/3\left(\alpha_{xx}^G + \alpha_{yy}^G + \alpha_{zz}^G\right)$, $\alpha_{\perp}^G = 1/2\left(\alpha_{xx}^G + \alpha_{yy}^G\right)$, $\alpha_{\parallel}^G = \alpha_{zz}^G$ and $\langle\cos^2(\theta)\rangle = \frac{1}{MN}\sum_{j=1}^{MN}\cos^2(\theta_j)$ is calculated from the MD trajectories. As $\Delta\alpha^G = \alpha_{\parallel}^G - \alpha_{\perp}^G$ is small compared to $\hat{\alpha}^G$ for all cases considered here, we can approximate

$$\Delta n(t) \approx \frac{4\pi N}{V n_0}\Delta\alpha^G\left(\langle\cos^2(\theta)\rangle(t) - \frac{1}{3}\right) \tag{30}$$

with $n_0 = \sqrt{1 + 4\pi(N/V)\hat{\alpha}^G}$.

The effect of the THz pump on the molecular orientation and alignment

Figure 2 in the main text presents $\langle\cos(\theta)\rangle(t)$, a measure for the molecular orientation due to the applied THz pump pulse. Here, θ is the angle of the molecular dipole with respect to the polarization axis of the field. As expected, $\langle\cos(\theta)\rangle(t)$ is 0 before the pulse. In the presence of the field, the permanent dipoles of water start reorienting along the direction of the field. The maximum orientation of the molecules is achieved with a time lag of a few hundred fs in comparison to the electric field profile of the pulse. A similar effect can also be observed for the molecular alignment described by $\langle\cos^2(\theta)\rangle(t)$ (see Figure 2). The employed THz pulse in the frequency range of 0.3 - 3 THz couples to the collective modes of water connected by hydrogen bonds[19,20] and, as the field amplitude increases, the hydrogen bond network is weakened enough to allow for orientation and alignment. As soon as the electric field changes the polarization direction, the water molecules also respond and change their preferred orientation, which can be seen from the slight negative part of $\langle\cos(\theta)\rangle(t)$ towards the end of the pulse (see Figure 2).

Kerr Effect

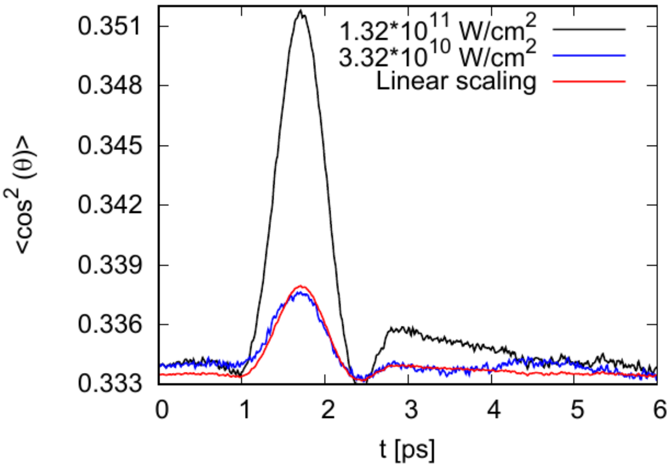

Supplementary Figure 9: The dependence of $\langle\cos^2(\theta)\rangle(t)$ on the pulse intensity I for the cases of I=1.33×10^{11} W/cm^2 (black) and I=3.32×10^{10} W/cm^2 (blue). A linear scaling of $\langle\cos^2(\theta)\rangle(t)$ with the field intensity and an equilibrium value of 1/3 can be anticipated. This linear relationship is employed to scale the results from I = 1.33×10^{11} W/cm^2 to I = 3.32×10^{10} W/cm^2 (red). The good agreement of the scaled data with the simulation result at I = 3.32×10^{10} W/cm^2 (blue) confirms the linear scaling.

The results presented in Supplementary Figure 9 employed a peak electric field amplitude that was ten times higher than the experimental electric field amplitude, which corresponds to a hundred times higher intensity in the simulations than the experiments. To obtain reliable Kerr effect data at the experimental intensity, the anticipated linear scaling of $\langle\cos^2(\theta)\rangle(t)$ with the field intensity was exploited. To test the linear scaling of $\langle\cos^2(\theta)\rangle(t)$ with the field intensity, simulations at different intensities were performed (see Supplementary Figure 9). Results obtained at a higher intensity (I=1.33×10^{11} W/cm^2) were then scaled down to a lower intensity (I = 3.32×10^{10} W/cm^2) employing a linear relationship of $\langle\cos^2(\theta)\rangle(t)$ with the field intensity and an equilibrium (i.e., with no field applied) value of 1/3. The scaled results were in very good agreement with results obtained from simulations at I = 3.32×10^{10} W/cm^2. This confirms the assumed linear scaling in the intensity regime considered in this work. Additionally, a linear scaling of the experimentally observed $\Delta\phi$ with employed field strength squared was also found (see Supplementary Figure 5).

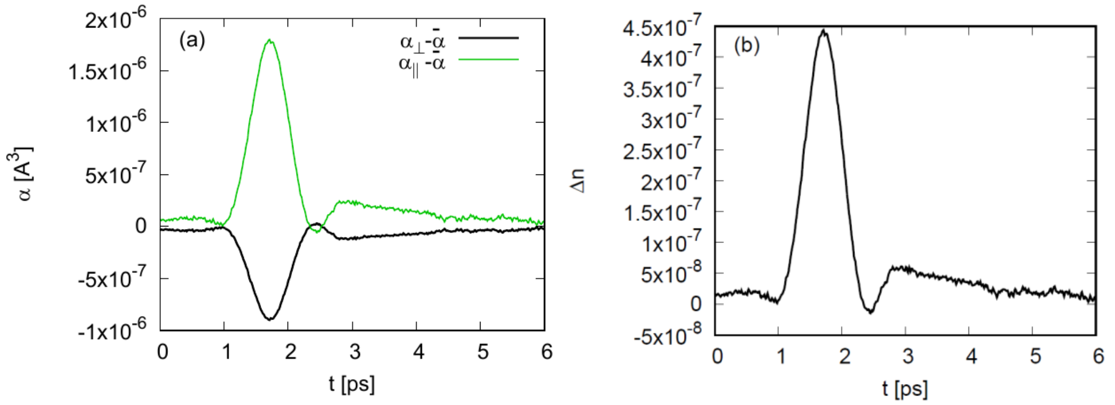

Supplementary Figure 10: Time-dependent parallel $\langle\alpha_{\parallel}\rangle$ and perpendicular $\langle\alpha_{\perp}\rangle$ polarizabilities of the water system at the experimental pulse intensity (Supplementary equation 28). (b) Time-dependent Δn due to the employed THz pump pulse (Supplementary equation 30).

The linear scaling of $\langle\cos^2(\theta)\rangle(t)$ with the field intensity was then employed to obtain the averaged polarizabilities at the experimentally employed field intensity. Supplementary Figure 10 presents the time-dependent averaged polarizabilities parallel $\langle\alpha_{\parallel}\rangle(t)$ and perpendicular $\langle\alpha_{\perp}\rangle(t)$ to the THz field polarization (panel (a)), $\Delta n(t)$ calculated using Supplementary equations (30) and (26) (panel (b)). The simulation model does not include any explicit electronic contributions and therefore only the molecular contribution to Δn was obtained. Comparing the Kerr effect obtained from the MD simulations with the molecular Kerr effect (Figure 1 of the main text) obtained by the fitting procedure described in the main text, a difference in sign as well as an order of magnitude difference in the strength of the effect is found. Given the form of $\Delta n(t)$ in Supplementary equation (30), the overestimation of the effect might arise due to inaccuracies in n_0, $\Delta\alpha^G$ or $\langle\cos^2(\theta)\rangle(t)$. An overestimation of $\langle\cos^2(\theta)\rangle(t)$ might arise due to overestimating the average field strength in the experimental sample. Given the α^G employed in this model, a value for n_0 of 1.27 is obtained, which reasonably well agrees with the experimental value of 1.33. Remaining differences might be due to induced effects of that change $\hat{\alpha}$ in the liquid system as well as vibrational effects not included in the model presented, but cannot account for the order of magnitude difference seen for $\Delta\varphi$. This would imply that the biggest error arises due to errors in the magnitude of $\Delta\alpha^G$ employed in our model. However, as discussed in the main text, the calculation of $\Delta\alpha^G$ is very hard as $\Delta\alpha^G/\hat{\alpha}^G$ is a tiny quantity and water seems nearly isotropic in terms of $\alpha_{\parallel}$ and $\alpha_{\perp}$. Thus, small errors in the calculation of α^G can result in large errors in the calculation in $\Delta\alpha^G$ and thus also in larger errors in the calculated $\Delta\varphi$ presented here. Furthermore, the magnitude of $\Delta\alpha$ might also be influenced by induced effects due to the surrounding liquid. The difference in the sign can also be rationalized by the limitation of the model that employs the gas-phase values and does not allow for any induced effects. As the water molecules align due to the THz pulse, the sign of the induced Kerr effect obtained from the MD simulations is given by $\Delta\alpha^G = \alpha_{\parallel}^G - \alpha_{\perp}^G > 0$. As pointed out above and in the main text, the accurate determination of $\Delta\alpha^G$ is very hard. A different choice of gas-phase polarizability, e.g., calculated from coupled cluster theory $\alpha^G = \begin{pmatrix} 1.375 & 0 & 0 \\ 0 & 1.442 & 0 \\ 0 & 0 & 1.321 \end{pmatrix}$ Å^3, $\Delta\alpha^G < 0$, [11] would result in a negative sign in the simulated Kerr effect in agreement with the experiment. In addition, the model employed neglects any effect on the polarizability of each molecule induced by its surrounding. As the magnitude of $\Delta\alpha^G$ is small, it seems possible that the induced effects may change its sign. The analysis based on the model presented here implies that the effective polarizability of a water molecule in liquid water has a small and negative polarizability

anisotropy $\Delta\alpha$. Comparing the timescale of the molecular Kerr effect in Figure 1 of the main text with the simulations, a similar time delay of the maximal response with respect to the pump pulse can be observed. Yet, the molecular contribution to $\Delta\varphi$ shows a longer tail in the experimental data than in the MD simulations. This difference may be attributed to too weak water-water interactions in the force field[15,21] that allow for a faster response of the water molecules to the sign change of the THz electric field. In addition, the alignment observed in the MD simulations is likely underestimated, which will also lead to a faster decay to equilibrium. It was found previously that rigid non-polarizable force fields underestimate the low-frequency IR bands of water compared to experimental results[22–24] as well as the dielectric constant[15] and, thus, the coupling of the employed water model to the applied THz pulse may be too weak.

Supplementary Note 6: The impact of temperature on the TKE of water

Supplementary Figure 11 shows the TKE of water in a cuvette, measured at various temperatures between 23 and 68 °C. Due to the temperature-independence of the electronic Kerr effect, the temperature-dependent signal $-d\Delta\phi(t)/dT$ originates from the molecular Kerr effect in water only. The resulting (blue) curve in Supplementary Figure 11 is in good agreement with the molecular contribution to the Kerr effect derived from the modelling of experimental data (black curve). The experimental setup was the same as described previously.

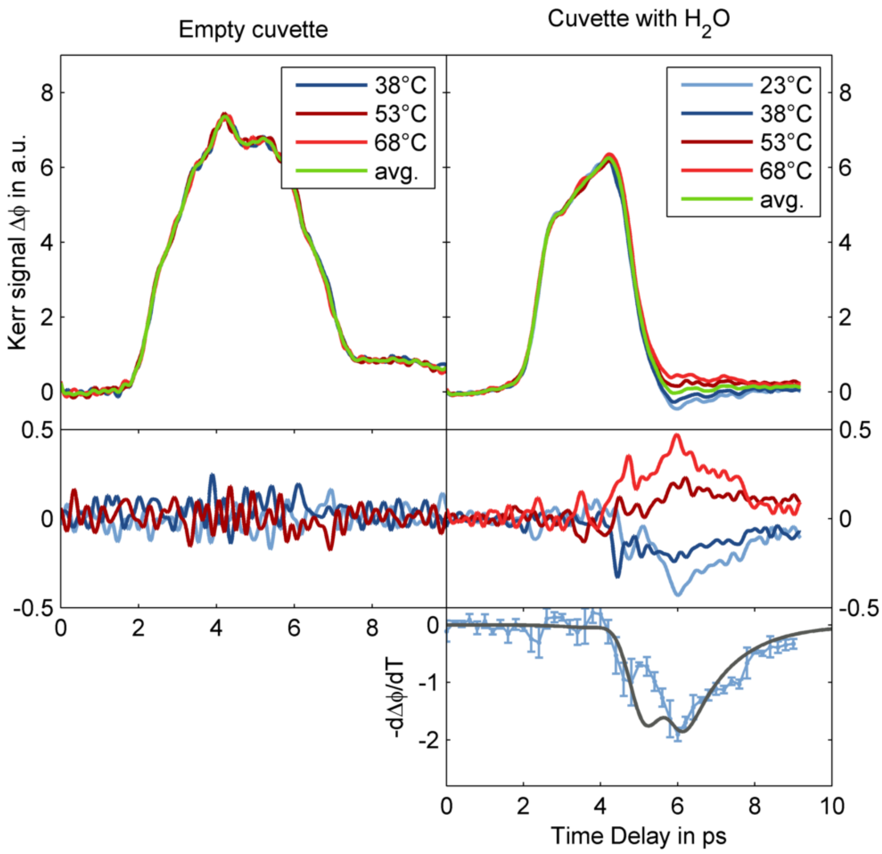

Supplementary Figure 11: Temperature-dependence of the TKE from an empty cuvette (left panels) and from water inside the cuvette (right panels) with temperatures given in the legend. The two middle panels show the difference of each curve and their average (green curve). While the purely electronic signal from the cuvette is temperature-independent, the TKE from water shows a clear temperature-dependence. According to the model in

equations 3&4 of the main text, the molecular contribution decreases with increasing temperature. The increase of the overall signal level observed here confirms that the molecular contribution must be negative. $-d\Delta\phi(t)/dT$ corresponds to the linearized temperature-dependence of the signal.

Supplementary References

1. Kalmykov, Y. P. Matrix method calculation of the Kerr effect transient and ac stationary responses of arbitrary shaped macromolecules. *J. Chem. Phys.* **131,** 074107 (2009).
2. Coffey, W. T. & Kalmykov, Y. P. *The Langevin Equation*. (World Scientific Series in Contemporary Chemical Physics, 2012).
3. Varshalovich, D. A., Moskalev, A. N. & Khersonskii, V. K. *Quantum Theory of Angular Momentum*. (World Scientific, 1988).
4. Wegener, W. A. Transient electric birefringence of dilute rigid-body suspensions at low field strengths. *J. Chem. Phys.* **84,** 5989 (1986).
5. Böttcher, C. J. F. & Bordewijk, P. *Theory of Electric Polarization, Vol II Dielectrics in Time-Dependent Fields*. (Elsevier, 1996).
6. Chevrot, G., Hinsen, K. & Kneller, G. R. Model-free simulation approach to molecular diffusion tensors. *J. Chem. Phys.* **139,** 154110 (2013).
7. Rozmanov, D. & Kusalik, P. G. Transport coefficients of the TIP4P-2005 water model. *J. Chem. Phys.* **136,** 044507 (2012).
8. O'Reilly, D. E. Self-diffusion coefficients and rotational correlation times in polar liquids. VI. Water. *J. Chem. Phys.* **60,** 1607 (1974).
9. Svishchev, I. M. & Kusalik, P. G. Dynamics in liquid water, water-d2, and water-t2: a comparative simulation study. *J. Phys. Chem.* **98,** 728 (1994).
10. Chelli, R., Pagliai, M., Procacci, P., Cardini, G. & Schettino, V. Polarization response of water and methanol investigated by a polarizable force field and density functional theory calculations: Implications for charge transfer. *J. Chem. Phys.* **122,** 074504 (2005).
11. Ito, H., Jo, J.-Y. & Tanimura, Y. Notes on simulating two-dimensional Raman and terahertz-Raman signals with a full molecular dynamics simulation approach. *Struct. Dyn.* **2,** 054102 (2015).
12. Avila, G. Ab initio dipole polarizability surfaces of water molecule: Static and dynamic at 514.5nm. *J. Chem. Phys.* **122,** 144310 (2005).
13. Gubskaya, A. V. & Kusalik, P. G. A mean-field approach for the determination of the polarizabilities for the water molecule in liquid state. *J. Comput. Methods Sci. Eng.* **4,** 641 (2004).
14. Gregory, J. K., Clary, D. C., Liu, K., Brown, M. G. & Saykally, R. J. The Water Dipole Moment in Water Clusters. *Science* **275,** 814 (1997).
15. Abascal, J. L. & Vega, C. A general purpose model for the condensed phases of water: TIP4P/2005. *J. Chem. Phys.* **123,** 234505 (2005).
16. Plimpton, S. Fast Parallel Algorithms for Short-Range Molecular Dynamics. *Journal of Computational Physics* **117,** 1 (1995).
17. Nosé, S. An extension of the canonical ensemble molecular dynamics method. *Mol. Phys.* **57,**

187 (1986).

18. Ryckaert, J. P., Ciccotti, G. & Berendsen, H. J. C. Numerical integration of the cartesian equations of motion of a system with constraints: molecular dynamics of n-alkanes. *J. Comput. Phys.* **23,** 327 (1977).

19. Heyden, M. *et al.* Dissecting the THz spectrum of liquid water from first principles via correlations in time and space. *Proc. Natl. Acad. Sci.* **107,** 12068 (2010).

20. Heyden, M. *et al.* Understanding the origins of dipolar couplings and correlated motion in the vibrational spectrum of water. *J. Phys. Chem. Lett.* **3,** 2135 (2012).

21. Zielkiewicz, J. Structural properties of water: Comparison of the SPC, SPCE, TIP4P, and TIP5P models of water. *J. Chem. Phys.* **123,** 104501 (2005).

22. Guillot, B. A molecular dynamics study of the far infrared spectrum of liquid water. *J. Chem. Phys.* **95,** 1543 (1991).

23. Sonoda, M. T., Vechi, S. M. & Skaf, M. S. A simulation study of the optical Kerr effect in liquid water. *Phys. Chem. Chem. Phys.* **7,** 1176 (2005).

24. Torii, H. Cooperative contributions of the intermolecular charge fluxes and intramolecular polarizations in the far-infrared spectral intensities of liquid water. *J. Chem. Theory Comput.* **10,** 1219 (2014).
